# Here and there: the double-side transgene localization

**DOI:** 10.18699/VJ21.068

**Published:** 2021-10

**Authors:** P.A. Salnikov, A.A. Khabarova, G.S. Koksharova1, R.V. Mungalov, P.S. Belokopytova, I.E. Pristyazhnuk, A.R. Nurislamov, P. Somatich, M.M. Gridina, V.S. Fishman

**Affiliations:** Institute of Cytology and Genetics of the Siberian Branch of the Russian Academy of Sciences, Novosibirsk, Russia; Novosibirsk State University, Novosibirsk, Russia; Institute of Cytology and Genetics of the Siberian Branch of the Russian Academy of Sciences, Novosibirsk, Russia; Institute of Cytology and Genetics of the Siberian Branch of the Russian Academy of Sciences, Novosibirsk, Russia; Novosibirsk State University, Novosibirsk, Russia; Institute of Cytology and Genetics of the Siberian Branch of the Russian Academy of Sciences, Novosibirsk, Russia; Institute of Cytology and Genetics of the Siberian Branch of the Russian Academy of Sciences, Novosibirsk, Russia; Novosibirsk State University, Novosibirsk, Russia; Institute of Cytology and Genetics of the Siberian Branch of the Russian Academy of Sciences, Novosibirsk, Russia; Institute of Cytology and Genetics of the Siberian Branch of the Russian Academy of Sciences, Novosibirsk, Russia; Novosibirsk State University, Novosibirsk, Russia; Institute of Cytology and Genetics of the Siberian Branch of the Russian Academy of Sciences, Novosibirsk, Russia; Institute of Cytology and Genetics of the Siberian Branch of the Russian Academy of Sciences, Novosibirsk, Russia; Institute of Cytology and Genetics of the Siberian Branch of the Russian Academy of Sciences, Novosibirsk, Russia; Novosibirsk State University, Novosibirsk, Russia

**Keywords:** transgenesis, genome-wide screening, transgene mapping, sleeping beauty transposon, трансгенез, полногеномный скрининг, локализация трансгена, транспозон «Спящая красавица»

## Abstract

Random transgene integration is a powerful tool for developing new genome-wide screening approaches. These techniques have already been used for functional gene annotation by transposon-insertion sequencing, for identif ication of transcription factor binding sites and regulatory sequences, and for dissecting chromatin position effects. Precise localization of transgenes and accurate artifact f iltration are essential for this type of method. To date, many mapping assays have been developed, including Inverse-PCR, TLA, LAM-PCR, and splinkerette PCR. However, none of them is able to ensure localization of both transgene’s f lanking regions simultaneously, which would be necessary for some applications. Here we proposed a cheap and simple NGS-based approach that overcomes this limitation. The developed assay requires using intentionally designed vectors that lack recognition sites of one or a set of restriction enzymes used for DNA fragmentation. By looping and sequencing these DNA fragments, we obtain special data that allows us to link the two f lanking regions of the transposon. This can be useful for precise insertion mapping and for screening approaches in the f ield of chromosome engineering, where chromosomal recombination events between transgenes occur in a cell population. To demonstrate the method’s feasibility, we applied it for mapping SB transposon integration in the human HAP1 cell line. Our technique allowed us to eff iciently localize genomic transposon integrations, which was conf irmed via PCR analysis. For practical application of this approach, we proposed a set of recommendations and a normalization strategy. The developed method can be used for multiplex transgene localization and detection of rearrangements between them.

## Abstract (ru)

Полногеномные скрининговые методы, основанные на случайной интеграции экзогенных генетических конструкций, – новейший класс инструментов, открывающий возможности для изучения широкого спектра геномных процессов. Данный подход уже был применен к функциональному аннотированию генов млекопитающих, скринингу приспособленности бактерий, определению сайтов связывания факторов транскрипции, идентификации регуляторных генетических элементов и исследованию хромосомного эффекта положения. Все эти эксперименты требуют точной локализации трансгенов в геноме. Существующие на сегодняшний день методы картирования, такие как Inverse-PCR, TLA, splinkerette PCR и LAM-PCR, не позволяют одновременно определять оба участка генома, фланкирующие одну интеграцию трансгена, что ограничивает применимость подходов, в том числе связанных с хромосомной инженерией. В настоящей работе мы предлагаем метод, с помощью которого можно преодолеть это ограничение. Разработанная технология основана на фрагментации геномной ДНК, не затрагивающей интеграции трансгена. Это достигается путем исключения из последовательности вектора сайтов узнавания одного или нескольких ферментов рестрикции. Затем, как и в Inverse-PCR, были закольцованы молекулы лигированием в разбавленной смеси и секвенированы. Полученные данные дают возможность с высокой точностью идентифицировать перестройки и отделить их от артефактов лигирования, и, кроме того, отследить события транслокаций между интеграциями трансгенов. Это может быть использовано в экспериментах по изучению индуцируемых хромосомных перестроек. Для доказательства применимости метода мы с его помощью картировали интеграции транспозона Sleeping Beauty в клетки человека линии Hap1. Картированные интеграции были валидированы с помощью ПЦР-анализа. В статье приведен ряд рекомендаций для практического использования этого метода в экспериментах по множественной локализации интеграций трансгенных конструкций

## Introduction

Genome-wide screening assays are important tools for modern genetics and genomics. Many of these methods rely on the integration of exogenous sequences in unknown or random genomic regions, mostly via retroviral or transposon vectors. This approach has already been used for functional gene annotation by transposon-insertion sequencing (Deutschbauer et al., 2011; Goodman et al., 2011; Goh et al., 2017; Cain et al., 2020), for transcription factor binding sites (Wang et al., 2012; Moudgil et al., 2020) and regulatory sequences identification (Pindyurin et al., 2015), and for chromatin position effects dissection (Akhtar et al., 2013).

For all of these techniques, accurate localization of transgene integration sites is crucial. There are several well-established methods for massive parallel genomic mapping of integration sites, from Nanopore (Li et al., 2019; Nicholls et al., 2019) or whole-genome Next Generation Sequencing (NGS) (Zhang et al., 2012; Zastrow-Hayes et al., 2015; Park et al., 2017) to cheaper target PCR-mediated approaches, including Inverse-PCR (Akhtar et al., 2013), LAM-PCR (Gabriel et al., 2014; Wang et al., 2016), splinkerette PCR (Friedrich et al., 2017), and TLA (de Vree et al., 2014; Laboulaye et al., 2018).

Importantly, current NGS-based methods cannot capture both transgene-flanking regions (5′ and 3′) simultaneously. Double-side localization is useful for artifact filtration and detection of translocation events occurring during the integration process, which could confound certain experiments (Francke et al., 1992). Furthermore, this is useful for screening approaches in the field of chromosome engineering, where chromosomal recombination events between transgenes occur in a cell population, such as Scramble technique (Dymond, Boeke, 2012; Hochrein et al., 2018) and others (Smith et al., 1995; Uemura et al., 2010). Conventional Inverse-PCR, routinely employed for transgene insertion identification, is unable to differentiate cases of normal insertion and exchange of flanking regions between different integrations in multiplex analysis. Despite its rarity in the standard conditions, a number of developing methods requires a precise detection of these events. Our approach provides double-sided transgene localization that can be applied for translocation detection between transgene integration points.

Here we developed a cheap Inverse-PCR-based approach enabling us to link 5′ and 3′ transposon flanking regions for all integration sites simultaneously. To demonstrate the method’s feasibility, we applied it for mapping SB transposon integration in the human HAP1 cell line. Our technique allowed us to efficiently localize genomic transposon integrations. For practical application of this approach, we suggested a set of recommendations and a normalization strategy. The developed method can be used for multiplex transgene localization and detection of rearrangements between them.

## Materials and methods

Plasmid vectors. The Sleeping Beauty transposon vector pSB_LoxP was generated via Gibson Assembly (NEB) by amplifying ITR sites from pSBbi-GP (Addgene #60511) and LoxP-rtTA sequence from pLeGO-rtTA (kind gift from Dr. A.M. Yunusova) and integrating into the pJET 1.2 vector (ThermoFisher, USA). We used PCR-mediated mutagenesis to substitute C to G in the CATG sequence within the right ITR (FaeI site). This resulted in the vector used for genomic transposon integration.

A vector expressing SB100X transposase was from Addgene (#34879). To allow selection of transposase-expressing cells, IRES-GFP cassette was amplified from vector Cre- IRES-PuroR (Addgene #30205) and inserted between transposase coding sequence and polyA signal using NEB Gibson Assembly, resulting in pSB100X-GFP vector.

Cell culture and Neon transfection. HAP1 cells were cultured in IMDM with 10 % FBS and 1xPen/Strep (Gibco, USA) according to manufacturer recommendations. Fluorescenceactivated cell sorting (FACS) and subcloning was performed on BD FACSAria™ III sorter on 96-well plates or manually. Transfections were done on the Neon transfection system under the following conditions: 1400 V, 20 ms 1 pulse and 1.5 μg total plasmid DNA (transposase expression and transposon carrying plasmid ratio 1:4). On day 2 after transfection, we performed a cell sorting of GFP positive cells.

PCR. For PCRs, qPCR and Double-side Inverse-PCR genomic DNA was extracted using a standard phenol-chloroform extraction protocol. All PCR procedures were performed using the PCR with Taq enzyme (#M0267 NEB, USA) and specific primers (available on request). qPCR was performed using BioMaster HS-qPCR (2×) (MN020-2040 Biolabmix, Russia) kit with specific primers and FAM-BHQ1 probe (qpcr_M2RTta_F: AGACTGGACAAGAGCAAAGT; qpcr_M2RTta_R: TTGAGCAGCCTACCCTGT; qpcr_ M2RTta_probe: FAM-TCGAAGGCCTGACGACAAGGABHQ; qpcr_Syn1_F CCCAAATACCAGGCAACCCA, qpcr_Syn1_R GGAAGGGGCTCAACAGTAGG, qpcr_ Syn1_probe: FAM-TTGGTCCCAAATCTCTCCAGCACABHQ). To allow absolute quantification, plasmid vector containing transposon and SYN1 PCR fragments was constructed and used for normalization. The data was analyzed using 2ddCt methods implemented in QuantStudio v1.3 software (Applied Biosistems, USA).

Double-side Inverse-PCR sequencing library preparation. DNA was isolated from cell pellet by phenol-chloroform extraction. DNA was digested overnight in 50 μl reaction at 37 °C by 5U NlaIII isoschizomer FaeI (E495 SibEnzyme, Russia) in final DNA concentration 100 ng/μl. Enzyme was inactivated by incubation at 65 °C for 10 min and 500 ng of digested DNA was ligated using T4 DNA ligase (E319, SibEnzyme) at 4 °C overnight in 100 μl reaction volume. 1 μl of ligation mix was used in PCR with Taq polymerase (#M0267 NEB, USA) (annealing 60 °C, elongation 3 min, 40 cycles). PCR products were diluted 100-fold and 1 μl was used in the next round of nested PCR. PCR products were analyzed by agarose gel electrophoresis and either used in the third round of nested PCR or purified using AMPure XP beads (A63882 Beckman Coulter, USA). 1 ng of purified PCR product was used for NGS adaptor ligation (SeqCap Adapter Kit B, 07141548001 Roche, Switzerland) using KAPA HyperPrep kit (07962363001 Roshe) according to manufacturer protocol with 15 cycles of post-ligation PCR.

NGS data analysis. We demultiplexed reads originating from different NGS-libraries based on barcode sequences (barcode_seq) included in 5′-end of primers using cutadapt (Martin, 2011) with –g barcode_seq –G barcode_seq –overlap 6 –e 0. Next, primers (primer_seq) were removed using cutadapt with parameters –g primer_seq –G primer_seq –overlap 20. Processed reads were aligned to human genome hg38 using bwa mem with default parameters (Li, Durbin, 2009). Regions covered by at least one read were found using bedtools genomecov (Quinlan, Hall, 2010) and a homemade python script. In addition, every covered region was manually analyzed in Integrated Genome Browser (IGV) (Robinson et al., 2011), which allowed distinguishing insertions sites from random ligations and other artifacts. The following analysis of rearrangements was done using homemade python scripts that counted the number of reads with mates or supplementary alignments in different insertion sites.

## Results and discussion

We improved a standard inverse-PCR assay for efficient localization of transgene integration. A key aspect of this strategy is the ability to recognize simultaneously both 5′ and 3′ flanking regions of transgenes in a multiplex NGS-based assay. As in conventional inverse-PCR, our assay consists of five steps: 1) DNA fragmentation, 2) ligation under low DNA concentration conditions, which favors circularization, 3) nested-PCR using primer pairs annealing to the ends of transgenic sequence in outward orientation, 4) PCR products sequencing, and 5) computational analysis.

**Fig. 1.**
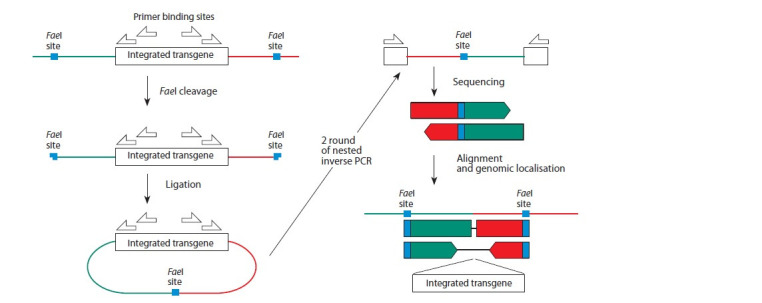
Conceptual scheme of Inverse-PCR-based strategy allowing double-side detection of transgene integration sites Genomic DNA carrying transgene integration sequentially are fragmented, re-ligated, PCRed from nested primers and obtained products are sequenced. Resulting reads alignment forms recognizable patterns depending on integration point and fragmentation sites position.

In our modification (Fig. 1), we propose to fragment DNA by restriction enzyme (RE), which recognizes sites that are absent in the transgene sequence. This results in the generation of DNA fragments, containing transposon and both 5′ and 3′ flanking regions up to the first RE recognition site in length. A subsequent ligation reaction generates circular molecules, which enables us to proceed with nested inverse-PCRs. PCR products are then used for NGS library preparation and paired-end sequencing. Reads are next trimmed from primers and transgenic sequences and aligned to the reference genome, which produces a recognizable pattern, defining integration sites and allowing to distinguish them from artifacts.

To prove this concept, we chose to use the pSB_LoxP plasmid previously constructed in our laboratory as a sleeping beauty (SB) transposon-containing vector. It contains a short LoxP-sequence cloned between two SB inverse terminal repeats (ITRs), and via transposase-mediated integration generates 833 bp long DNA inserts. Although 4-bp cutting RE is preferable for effective Inverse-PCR library construction, it is hardly possible to find at least one RE that does not cleave the integrating DNA. However, we noted that the transposon sequence contains a single FaeI (NlaIII) recognition site within ITR sequence. To disrupt this site, we introduced single nucleotide substitution by site-directed mutagenesis. This allowed us to employ FaeI as RE for DNA fragmentation in integration localization assay.

To test this approach, we co-transfected human HAP1 cells with the developed transposon-containing plasmid and the SB100X vector expressing transposase and GFP proteins, followed by cell sorting of the GFP-positive cells the next day. Five days after transfection the GFP-negative cells were subcloned using FACS. This ensured the loss of the transposaseexpressing plasmid and excluded the possibility of continuous “jumping” of the transposons across the genome. Two of the obtained subclones were randomly picked to proceed with localization assay.

For these clones, we constructed and sequenced an Inverse PCR NGS library following the approach described above. We obtained ~300 000 read pairs for the first and ~200 000 for the second clone. NGS data analysis suggested 73 and 13 integration site candidates (regions covered by at least ten reads) for each clone respectively. Every covered region was manually analyzed in IGV genome browser to distinguish insertion sites from random ligations and other artifacts. This analysis allowed us to identify 12 transposon insertions in the first clone and 6 insertions in the second clone. The rest were identified as artifacts generated on ligation step and supplementary alignment regions for transposons integrated into repetitive DNA elements.

**Fig. 2.**
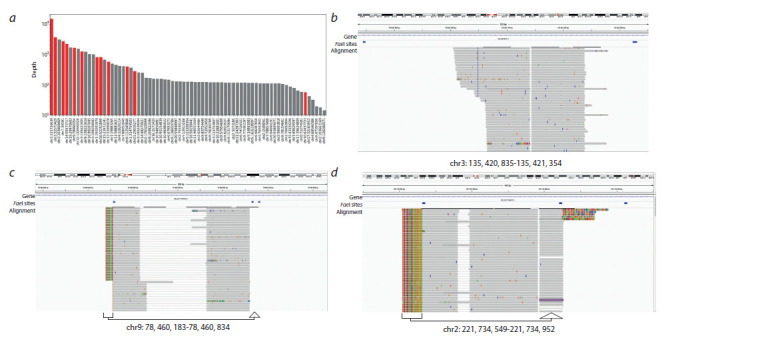
Transposon integrations analysis. a – bar plot representation of sequencing depth ( y, log scale) among integration candidate sites (x) for the first subclone. Red bars represent bona fide integrations, gray – ligation artifacts. Bona f ide integrations were discriminated based on the manual curation of NGS results in the IGV browser and conf irmed using PCR (see the text for details); b–d – IGV screenshots showing read alignments for some integrations. Arrows underneath represent supplementary sequences or softclipped read bases corresponding to the bases transferred from another transgene f lanking region through the FaeI site.

To obtain an estimate of the number of integrations using an orthogonal approach, we employed qPCR strategy with internal plasmids control (see Materials and methods). We obtained approximately 20 insertions for the first clone and 10 for the second. Despite the possibly low estimation accuracy of qPCR method, this result can identify bias of insertion underrepresentation of our Inverse-PCR-based strategy. Moreover, coverage is not a sufficient parameter for integration site identification. As shown on Fig. 2, a, both high-represented artifacts and low-represented transposon integrations are observed, although bona f ide integration sites typically show higher coverage.

**Fig. 3.**
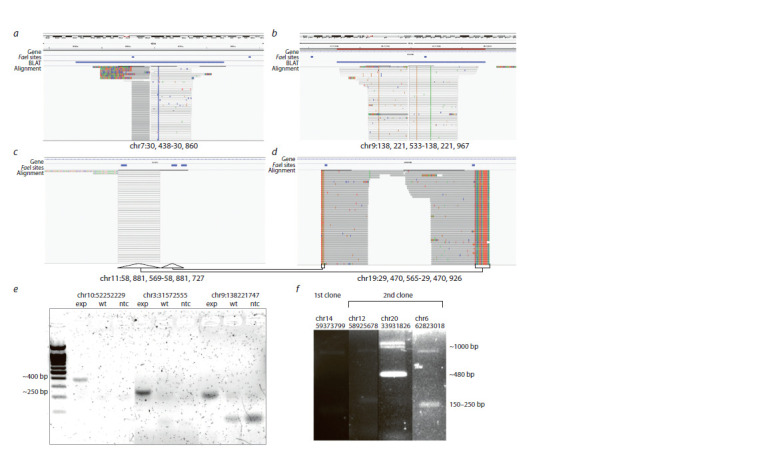
Transposon integrations analysis and PCR-conf irmation. a–d – IGV screenshots showing read alignments for some integrations (a–c) and ligation artifact (d). Arrows underneath represent supplementary sequences or soft-clipped read bases, corresponding to the bases transferred from another transgene flanking region through the FaeI site. e – one side PCR detection of transposon integration in the f irst clone. exp – experimental subclone with transposon integrations; wt – intact HAP1 cells; ntc – non-template control; f – double-side PCR integration detection. 1 kb product represents allele carrying transposon integration, whereas lower bands correspond to wild type allele product.

To dissect the nature of these biases, we investigated alignments individually. The typical expected pattern is shown on Fig. 2, b: both mates in the reads pair start at the same point (TA dinucleotide, the obligatory SB integration site) and extend divergently. Depending on RE sites position, this pattern can transform: if a read crosses the RE site involved in the DNA circularization, its alignment will be truncated at RE site position and continue again on the other side of transgene integration loci (see Fig. 2, c, d ). The situation is more complicated if two RE sites are close to integration on both sides. The distance between these sites may be smaller than read length, and in this case transposon sequence appears at the end of the read. The worst case is when transposon integration is in a repeated sequence, which results in multiple reads alignment and difficulties with precise localization (Fig. 3, a and b). We observed one integration located in a repeated sequence and also flanked by closely located FaeI sites. We managed to recognize its position only using complementary information accidentally produced due to unintended ligation of a random sequence to one of the transgene ends. Supplementary aligned bases of those reads revealed the bona f ide transposon integration point (see Fig. 3, c and d ).

Further, we decided to validate the integration sites determined by NGS-approach using conventional PCR. Six transposons were mapped within intergenic regions, and seven were integrated into gene introns. Since SB integration is semi-random and tends to occur in the active chromatin, this is not surprising. However, the genomic context of most integration sites in our subclones is low-complex. We picked a few integrations, the flanking regions of which allow us to design PCR primers. First, we confirmed integration events from one side using primer pairs, one of which was targeted to flanking regions and the other was placed in the transposon (see Fig. 3, e). Next, we obtained the PCR products containing the entire transposon with flanking sequences, using pairs of primers targeted to endogenous regions around the predicted integration site. This unambiguously confirmed the NGSbased localization results (see Fig. 3, f ).

## Conclusion

Summing up, we demonstrated the applicability of the proposed double-side transgene localization approach by successfully mapping 18 SB transposon integrations carrying an exogenic sequence. Our method provides simple detection of integration sites with confident artifact filtration via analysis of the read pair alignment pattern. Because the developed methods allow simultaneous detection of the flanking sequences at both transgene ends, we argue that this method can be used in future for genome-wide detection of transgene recombination events. Notably, the SB-transposon derived in this study contained a LoxP-site sequence, which makes it very simple to repurpose the developed system for induction of Cre-mediated chromosomal rearrangements.

of the developed method that we have to discuss. First, the proximity of chosen RE sites to transposon integration has a huge influence on the representation of integration site in the sequencing data. In this experiment we have already seen that too close RE sites complicate transposon mapping, whereas too distant sites can fully prevent PCR product generation. It happens because library preparation steps such as PCR and purification have a size-selecting manner. To solve this problem, one may develop new protocols where sonication is used for DNA fragmentation during library construction. Importantly, sonication will yield significantly higher noise for transgene recombination events detection due to the possibility to introduce DNA breaks inside the transgene. Second, in contrast to common inverse-PCR, our approach requires the choice of a RE that would not cut the transgene sequence. It is challenging even for short insertions, so we propose to introduce single-nucleotide substitutions into vector sequences. Third, neither our approach, nor other transgene localization methods ensure the identification of all integrations. For practical use, we recommend complementing our method with transgene insertions quantification via qPCR.

## Conflict of interest

The authors declare no conflict of interest.
